# Is antipsychotic polypharmacy associated with metabolic syndrome even after adjustment for lifestyle effects?: a cross-sectional study

**DOI:** 10.1186/1471-244X-11-118

**Published:** 2011-07-26

**Authors:** Fuminari Misawa, Keiko Shimizu, Yasuo Fujii, Ryouji Miyata, Fumio Koshiishi, Mihoko Kobayashi, Hirokazu Shida, Yoshiyo Oguchi, Yasuyuki Okumura, Hiroto Ito, Mami Kayama, Haruo Kashima

**Affiliations:** 1 Yamanashi Prefectural KITA Hospital, 3314-13 Kamijominamiwari, Asahi-machi, Nerasaki-shi, Yamanashi, Japan; 2 Faculty of Nursing, Yamanashi Prefectural University, 1-6-1 Ikeda, Koufu-shi, Yamanashi, Japan; 3 Department of Social Psychiatry, National Institute of Mental Health, National Center of Neurology and Psychiatry, 4-1-1 Ogawahigashi-machi, Kodaira-shi, Tokyo, Japan; 4 Psychiatric & mental Health Nursing, St. Luke's College of Nursing, 10-1 Akasi-cho, Chuo-ku, Tokyo, Japan; 5 Department of Psychiatry, Neuropsychiatry, Keio University, School of Medicine, 35 Shinano-machi, Shinjuku-ku, Tokyo, 160-8582, Japan

## Abstract

**Background:**

Although the validity and safety of antipsychotic polypharmacy remains unclear, it is commonplace in the treatment of schizophrenia. This study aimed to investigate the degree that antipsychotic polypharmacy contributed to metabolic syndrome in outpatients with schizophrenia, after adjustment for the effects of lifestyle.

**Methods:**

A cross-sectional survey was carried out between April 2007 and October 2007 at Yamanashi Prefectural KITA hospital in Japan. 334 patients consented to this cross-sectional study. We measured the components consisting metabolic syndrome, and interviewed the participants about their lifestyle. We classified metabolic syndrome into four groups according to the severity of metabolic disturbance: the metabolic syndrome; the pre-metabolic syndrome; the visceral fat obesity; and the normal group. We used multinomial logistic regression models to assess the association of metabolic syndrome with antipsychotic polypharmacy, adjusting for lifestyle.

**Results:**

Seventy-four (22.2%) patients were in the metabolic syndrome group, 61 (18.3%) patients were in the pre-metabolic syndrome group, and 41 (12.3%) patients were in visceral fat obesity group. Antipsychotic polypharmacy was present in 167 (50.0%) patients. In multinomial logistic regression analyses, antipsychotic polypharmacy was significantly associated with the pre-metabolic syndrome group (adjusted odds ratio [AOR], 2.348; 95% confidence interval [CI], 1.181-4.668), but not with the metabolic syndrome group (AOR, 1.269; 95%CI, 0.679-2.371).

**Conclusions:**

These results suggest that antipsychotic polypharmacy, compared with monotherapy, may be independently associated with an increased risk of having pre-metabolic syndrome, even after adjusting for patients' lifestyle characteristics. As metabolic syndrome is associated with an increased risk of cardiovascular mortality, further studies are needed to clarify the validity and safety of antipsychotic polypharmacy.

## Background

Metabolic syndrome is a cluster of metabolic dysfunctions, including central obesity, hypertension, glucose, and lipid abnormalities. Those with the syndrome have a two- to threefold increase in cardiovascular mortality and a twofold increase in all-cause mortality [1]. Patients with schizophrenia are more likely to have metabolic syndrome than the general population [2].

To date, a few research studies have reported an association between antipsychotic polypharmacy and metabolic syndrome [3,4]. Limited evidence currently exists regarding the benefits of antipsychotic polypharmacy, and antipsychotic monotherapy is consistently recommended in the treatment of patients with schizophrenia [5,6]. Antipsychotic polypharmacy is, however, commonplace in the treatment of schizophrenia [7-11], and has been reported to occur in a wide range (13-90%) of cases. In Japan, in particular, polypharmacy has been reported to occur at a higher rate than in other countries [12].

If antipsychotic polypharmacy, which is not recommended, is associated with a greater risk of metabolic syndrome, the spread of polypharmacy is a serious concern. However, it remains unclear among earlier studies whether antipsychotic polypharmacy is associated with metabolic syndrome as a direct result of patients' unhealthy lifestyle. Patients with schizophrenia are likely to make poor dietary choices, have low rates of physical activity, and smoke cigarettes [13], and their unhealthy lifestyle is assumed to be associated with an increased risk of metabolic syndrome. However, as little information is available on the association between metabolic syndrome and antipsychotic polypharmacy in conjunction with patients' lifestyle, further research is needed any such association.

In this cross-sectional study, we aimed to investigate the relationships between antipsychotic polypharmacy and metabolic syndrome in outpatients with schizophrenia, with adjustment for the effects of lifestyle.

## Methods

### Study participants

Participants who lived in the community and received psychiatric outpatient treatment were recruited from April 2007 to October 2007. The study inclusion criteria were: regular attendance at Yamanashi Prefectural KITA Hospital, Japan; an ICD-10 diagnosis of schizophrenia, schizotypal and delusional disorders; and age 18 years or older.

During the study period, of all 599 patients who fulfilled the inclusion criteria in this study, 399 consented to participate in the study. As 65 of these patients did not complete the questionnaire, data from 334 patients were used in the analysis.

The study design was approved by the Ethics Committees of Yamanashi Prefectural KITA Hospital. Written informed consent was obtained from all participants.

### Assessment

Assessment in this study consisted of sociodemographics (age, gender), duration of psychiatric treatment, family history of lifestyle-related disease, metabolic syndrome, prescribed antipsychotics, and participants' lifestyle. In addition, psychiatrists in charge of the participants assessed the patients on the Global Assessment of Functioning (GAF) scale.

### Metabolic syndrome

Rather than using the discrete diagnostic category of metabolic syndrome, we divided metabolic syndrome into four groups based on severity of metabolic disturbance (metabolic syndrome, pre-metabolic syndrome, visceral fat obesity and normal), since metabolic syndrome is continuously disturbed in nature [14]. In accordance with the diagnostic criteria proposed by the Japanese Committee of the Metabolic Syndrome Diagnostic Criteria [15], metabolic syndrome was defined as visceral fat obesity (abdominal circumference: ≥85 cm for males, ≥90 cm for females) and at least two of the following three criteria: elevated blood glucose (fasting glucose level ≥110 mg/dL), lipid abnormalities (triglycerides ≥150 mg/dL and/or high-density lipoprotein (HDL) cholesterol <40 mg/dL), and elevated blood pressure (systolic blood pressure ≥130 mmHg and/or diastolic blood pressure ≥85 mmHg). Current treatment with diabetes, lipid-lowering, or antihypertensive medication fulfilled the criterion for elevated blood glucose, lipid abnormality, and elevated blood pressure, respectively. Pre-metabolic syndrome was defined as the presence of one of the above three criteria in addition to visceral fat obesity.

We classified metabolic syndrome in the following four groups: the normal group did not fulfill the criteria of visceral fat obesity, the visceral fat obesity group fulfilled only the criteria of visceral fat obesity, the pre-metabolic syndrome group was defined by the presence of only one of the three criteria above in addition to visceral fat obesity, and the metabolic syndrome group was defined by the presence of at least two of the three criteria above in addition to visceral fat obesity. Participants were given written instructions to fast overnight on the day before assessment, and asked to confirm their fasting status before blood samples were taken. A single venous blood sample was withdrawn and analyzed for glucose, triglycerides, and HDL cholesterol. Nurses measured abdominal circumference and blood pressure.

### Prescribed antipsychotics

We investigated prescribed antipsychotics from patient charts on the day we measured the participant's metabolic syndrome parameters. All dosages of antipsychotic drugs were converted into chlorpromazine equivalents [16] in order to estimate the total daily chlorpromazine-equivalent dose.

In this study, polypharmacy was defined as the concomitant use of two or more antipsychotics, while monotherapy was defined as the use of only one antipsychotic.

Antipsychotic treatment in Japan was subject to special conditions during the study period. First, clozapine had not been launched at this time. Second, olanzapine and quetiapine were contraindicated for patients with diabetes or a history of diabetes because it was reported that some patients that were treated with olanzapine and quetiapine developed severe hyperglycemia and diabetic coma.

### Assessment of participants' lifestyle

We assessed the participants' dietary habits, physical activity, and smoking habits. With regards to dietary habits, these were assessed by an originally designed self-reporting questionnaire that consisted of the following four items, which have been used in earlier studies: snack eating (Do you eat snacks?), intake of fatty foods (Do you eat fatty foods?), preference for a high-salt diet (Do you put soy sauce or Worcestershire sauce on your food?), and consumption of soft drinks (Do you drink soft drinks?) [17,18]. Each item was scored on a 4-point scale (1 = never, 2 = rarely, 3 = sometimes, 4 = always).

To assess the participants' physical activity, we used the Exercise and Physical Activity Guide for Health Promotion 2006 [19]. In this guide, physical activity consists of exercise and non-exercise activities (e.g., walking, cleaning the floors, and walking up and down stairs). The units used to express the intensity and quantity of physical activity are "MET" and "MET × hour", respectively. MET is calculated as energy expenditure (oxygen uptake, mL/kg/min) during a specific physical activity divided by sitting/resting energy expenditure. Defining the MET of sitting/resting as 1, that of normal walking, for example, is 3. The unit "MET × hour" (expressed as "Ex" for Exercise (*Ekusasaizu *in Japanese)) was calculated by multiplying the MET by the duration of the activity (hour). The goal for physical activity was set at 23 Ex or more per week, with 3 MET of physical activity set as the minimum (cut off).

Using the Compendium of Physical Activities [19], we interviewed participants about their exercise and non-exercise activities with more than 3 MET in the one week prior to the study day, and calculated the quantity of their physical activity.

Participants' smoking habits were rated as 1 (= 21 or more cigarettes per day), 2 (= 6 to 20), 3 (= 1 to 5), or 4 (= no cigarette).

### Data analyses

Analyses of variance, chi-square tests, and Kruskal-Wallis tests were used to compare demographic, treatment and clinical variables in the classification of metabolic syndrome.

To examine the effects of antipsychotic polypharmacy on metabolic syndrome, we conducted multinomial logistic regression analyses, with the classification of metabolic syndrome as the dependent variable. For the analyses, we entered the variables whose p-values were less than 0.1 in univariate tests into the model. A p value of <0.05 was considered statistically significant. Data were analyzed using SPSS 14.0 J for Windows.

## Results

### Characteristics, lifestyle, and antipsychotic treatment of participants (Table 1)

**Table 1:** Characteristics, lifestyle and antipsychotic treatment in total participants and four groups

	Total (n = 334)	Normal (n = 158)	visceral fat obesity (n = 41)	pre-metabolic (n = 61)	Metabolic (n = 74)	p
*Characteristics*						
Age, mean (SD), y	44.2 (12.3)	43.5 (13.2)	42.4 (11.4)	43.4 (12.2)	47.2 (10.6)	0.11
Female, % (n)	42.8 (143)	62.7 (99)	22.0 (9)	21.3 (13)	29.7% (22)	<0.01
GAF, mean (SD)	53.5 (15.3)	54.6 (15.0)	51.3 (15.5)	53.3 (15.6)	52.5 (15.6)	0.58
Family history, % (n)	48.8 (163)	48.7 (77)	48.8 (20)	44.3 (27)	52.7 (39)	0.81
Duration of psychiatric treatment, mean (SD), y	18.2 (12.1)	16.8 (12.2)	18.7 (11.8)	17.2 (11.3)	21.5 (12.1)	0.04
						
*Lifestyle*						
Snacks eating, % (n)						0.39
1 = never	12.9 (43)	13.3 (21)	9.8 (4)	13.1 (8)	13.5 (10)	
2 = rarely	26.9 (90)	28.5 (45)	24.4 (10)	27.9 (17)	24.3 (18)	
3 = sometimes	40.7 (136)	39.2 (62)	31.7 (13)	41.0 (25)	48.6 (36)	
4 = always	19.5 (65)	19.0 (30)	34.1 (14)	18.0 (11)	13.5 (10)	
Fatty foods, % (n)						0.18
1 = never	2.4 (8)	5.1 (8)	0.0 (0)	0.0 (0)	0.0 (0)	
2 = rarely	21.6 (72)	23.4 (37)	17.1 (7)	23.0 (14)	18.9 (14)	
3 = sometimes	59.0 (197)	57.0 (90)	65.9 (27)	59.0 (36)	59.5 (44)	
4 = always	17.1 (57)	14.6 (23)	17.1 (7)	18.0 (11)	21.6 (16)	
High salt diet, % (n)						0.77
1 = never	4.5 (15)	3.2 (5)	1.6 (1)	1.6 (1)	10.8 (8)	
2 = rarely	20.1 (67)	22.2 (35)	19.5 (8)	19.7 (12)	16.2 (12)	
3 = sometimes	42.2 (141)	43.0 (68)	36.6 (15)	50.8 (31)	36.5 (27)	
4 = always	33.2 (111)	31.6 (50)	41.5 (17)	27.9 (17)	36.5 (27)	
Consumption of soft drink, % (n)						0.16
1 = never	11.1 (37)	13.3 (21)	9.8 (4)	11.5 (7)	6.8 (5)	
2 = rarely	21.6 (72)	26.6 (42)	29.3 (12)	9.8 (6)	16.2 (12)	
3 = sometimes	41.9 (140)	36.7 (58)	29.3 (12)	49.2 (30)	54.1 (40)	
4 = always	25.4 (85)	23.4 (37)	31.7 (13)	29.5 (18)	23.0 (17)	
Smoking habit, per day, % (n)						<0.01
1 = 21 or more	18.6 (62)	13.9 (22)	7.3 (3)	24.6 (15)	29.7 (22)	
2 = 6 to 20	21.6 (72)	17.7 (28)	26.8 (11)	24.6 (15)	24.3 (18)	
3 = 1 to 5	3.9 (13)	5.1 (8)	2.4 (1)	3.3 (2)	2.7 (2)	
4 = none	56.0 (187)	63.3 (100)	63.4 (26)	47.5 (29)	43.2 (32)	
						
Physical activity, mean (SD), Ex	22.4 (37.3)	21.0 (38.1)	17.9 (19.5)	30.0 (51.9)	21.7 (27.3)	0.34
						
*Antipsychotic treatment*						
Total daily dose, mean (SD), mg/d	596.6 (453.4)	510.3 (419.6)	769.2 (437.6)	668.4 (452.3)	626.2 (497.9)	<0.01
Antipsychotics contraindicated for diabetes, % (n)	35.0 (117)	62.7 (99)	65.9 (27)	63.9 (39)	56.8 (42)	0.74
Antipsychotic polypharmacy, % (n)	50.0 (167)	40.5 (64)	61.0 (25)	63.9 (39)	52.7 (39)	0.01

The mean age of the 334 participants was 44.2 years, and 42.8% were female. The mean GAF score was 53.5, 48.8% had a family history of lifestyle-related disease, and the mean duration of psychiatric treatment was 18.2 years. The mean value of physical activity was 22.4 Ex, and the mean score for smoking habit was 3.0.

The mean dose in chlorpromazine equivalents was 596.6 mg/day, and 35.0% received olanzapine and quetiapine. One hundred six (31.7%) patients received two antipsychotics, 48 (14.4%) patients were on three antipsychotics, and 13 (3.9%) patients were on four antipsychotics. According to the definition in this study that polypharmacy was the concomitant use of two or more antipsychotics, 167 participants (50.0%) were receiving antipsychotic polypharmacy.

### Category of metabolic syndrome

Of the 334 participants, 176 (52.7%) fulfilled the visceral fat obesity criteria, 92 (27.5%) fulfilled the elevated blood glucose criteria, 138 (41.3%) fulfilled the lipid abnormality criteria, and 105 (31.4%) fulfilled the elevated blood pressure criteria. Seventy-four (22.2%) patients were in the metabolic syndrome group, 61 (18.3%) patients were in the pre-metabolic syndrome group, 41 (12.3%) patients were in the visceral fat obesity group, and 158 (47.3%) were in the normal group. The characteristics, lifestyle and antipsychotic treatment in each group are summarized in Table 1. The rate of polypharmacy in the groups of metabolic syndrome, pre-metabolic syndrome, visceral fat obesity and normal were 52.7%, 63.9%, 61.0%, and 40.5%, respectively.

Compared to the monotherapy group, the polypharmacy group was more likely to fulfil the visceral fat obesity criterion (61.7% vs. 43.7%, p = 0.0014) and the elevated blood glucose criterion (32.9% vs. 22.2%, p = 0.037), and less likely to fulfil the elevated blood pressure criterion (26.3% vs. 36.5%, p = 0.045). The prevalence of the metabolic syndrome group in monotherapy and polypharmacy showed no significant difference (23.4% vs. 21.0%, p = 0.60). However, the polypharmacy group was more likely to be the pre-metabolic syndrome group (46.7% vs. 34.1%, p = 0.019).

### Multinomial logistic regression analyses (Table 2)

**Table 2:** Multinomial logistic regression analyses

	visceral fat obesity		premetabolic syndrome		metabolic syndrome	
**Variable**	**AOR**	**95% CI**	**AOR**	**95% CI**	**AOR**	**95% CI**

Gender (male)	**7.104**	**2.990-16.879**	**6.122**	**2.955-12.683**	**3.427**	**1.835-6.401**
Smoking habit, per day						
21 or more	0.353	0.093-1.337	1.726	0.750-3.974	**2.298**	**1.074-4.916**
6 to 20	0.882	0.357-2.183	1.103	0.483-2.521	1.537	0.714-3.308
1 to 5	0.480	0.054-4.286	0.784	0.144-4.266	0.736	0.143-3.799
none (reference)	1	--	1	--	1	--
Duration of psychiatric treatment, y	1.006	0.974-1.039	0.990	0.962-1.019	**1.028**	**1.003-1.054**
Total daily dose (10 mg units)	**1.011**	**1.003-1.019**	1.007	0.999-1.015	1.005	0.998-1.012
Antipsychotic polypharmacy	1.580	0.709-3.521	**2.348**	**1.181-4.668**	1.269	0.679-2.371

The dependent variable has four categories: normal, visceral fat obesity, pre-metabolic, and metabolic syndrome. The latter three categories are compared with the normal category.

AOR: adjusted odds ratio, CI: confidence interval

Nagelkere's R square = 0.26.

Multinomial logistic regression analyses revealed that the metabolic syndrome group was associated with being male, longer duration of psychiatric treatment, and heavier smoking habit. The pre-metabolic syndrome group was associated with being male and antipsychotic polypharmacy. The visceral fat obesity group was associated with being male and higher antipsychotic total daily dose.

Thus, overall, antipsychotic polypharmacy was not related to the severity of symptoms in the metabolic syndrome group but was related to the severity of symptoms in the pre-metabolic syndrome group.

## Discussion

Our study shows that antipsychotic polypharmacy is not correlated with metabolic syndrome but is correlated with the wider range of the syndrome when adjusting for the effects of lifestyle in outpatients with schizophrenia. These findings indicate that antipsychotic polypharmacy contributes in part to metabolic syndrome.

It remains unclear why antipsychotic polypharmacy is correlated with metabolic disturbance. Earlier studies suggested that various receptors effects, such as H_1_, D_2_, 5-HT_1A_, 5-HT_2C_, α_2_, and M_3_, might contribute to metabolic disturbance [20]. We speculate that the complex receptor-binding profiles of antipsychotic polypharmacy might be one of the causes of metabolic disturbance.

Among earlier studies, the association between metabolic syndrome and antipsychotic polypharmacy was not certain. For example, Correll et al. [3] observed that patients who receive antipsychotic polypharmacy had significantly higher rates of metabolic syndrome in univariate analyses, but antipsychotic polypharmacy was not independently associated with metabolic syndrome in logistic regression analyses which accounted for demographic and clinical variables. They speculated that antipsychotic polypharmacy was not a primary factor for metabolic syndrome, and that factors related to antipsychotic polypharmacy, such as inactivity, contributed to the risk of metabolic syndrome.

Physical activity was not associated with metabolic syndrome of any severity in this study. We infer that the association between metabolic syndrome and antipsychotic polypharmacy is not certain because of the effect of antipsychotic polypharmacy on lowering blood pressure, rather than because of the effect of inactivity. It was reported that polypharmacy was associated with a significantly higher drop in systolic pressure than monotherapy [21]. This might be due to the effects of a higher dose than that received during monotherapy or a drug interaction which created dopaminergic and noradrenergic deficiency conditions, such as Shy-Drager syndrome. Similarly, in the present study, patients receiving antipsychotic polypharmacy were less likely to fulfil the criterion of elevated blood pressure. Consequently, because antipsychotic polypharmacy tended not to be associated with elevated blood pressure, which is one of the three criteria for metabolic syndrome, polypharmacy may not have been correlated with metabolic syndrome, which needs to fulfil two or more of the three criteria, but rather with pre-metabolic syndrome, which needs to fulfil one or more of the criteria. We speculate that antipsychotic polypharmacy is directly associated with metabolic disturbance and increases the risk for metabolic syndrome, but the effect on lowering blood pressure masks the diagnosis of metabolic syndrome.

Another reason for our finding that polypharmacy contributes in some way to metabolic syndrome is that psychiatrists might be reluctant to prescribe additional antipsychotics for patients with metabolic syndrome to avoid worsening their metabolic profiles; however, for patients with pre-metabolic syndrome, they might not hesitate to prescribe additional antipsychotic.

Antipsychotic polypharmacy was not significantly associated with the visceral fat obesity group. That may be why the sample size in the group was small. We speculate that the association between polypharmacy and the visceral fat obesity may become significant if the sample size is larger.

Among the lifestyle factors, smoking habit was associated with prevalence of metabolic syndrome. It is considered to be an important risk factor for metabolic syndrome in the general population [22,23]. The prevalence of smoking in schizophrenia greatly exceeds that in the general population [24-26]. For the prevention of metabolic syndrome, it is necessary to provide guidance for lifestyle, such as smoking cessation advice, to patients with schizophrenia, especially those receiving antipsychotic polypharmacy.

The limitations of our study were a cross-sectional design, moderate sample size, high rate of refusal to participate in the study, and non-assessment of other psychotropic drugs except antipsychotics. In addition, special conditions were imposed on antipsychotic treatment in Japan at the time of the study, that is, clozapine had not yet been launched and olanzapine and quetiapine were contraindicated for patients with diabetes or a history of diabetes. Clozapine and olanzapine, in particular, are known as high-risk drugs for metabolic syndrome [27]. Therefore, the above special conditions are likely to have affected the results in this study.

## Conclusions

Our study is the first attempt to clarify the relationship between metabolic syndrome, antipsychotic polypharmacy, and patients' lifestyle. The findings indicate that antipsychotic polypharmacy, compared with monotherapy, may be independently associated with an increased risk of having pre-metabolic syndrome, even after adjusting for patients' lifestyle characteristics. Despite the fact that there is little evidence regarding the efficacy of antipsychotic polypharmacy in schizophrenia and that it is not recommended in its treatment of schizophrenia, it has been common practice in the past. As metabolic syndrome is associated with an increased risk of cardiovascular mortality, further studies are needed to clarify the validity and safety of antipsychotic polypharmacy in this patient population.

## Competing interests

The authors declare that they have no competing interests.

## Authors' contributions

FM participated in the design and the coordination of the study, performed the statistical analyses and drafted the manuscript. KS conceived of the study and participated in the design and the coordination of the study. YF, RM, FK, MiK, HS and YaO participated in the design and the coordination of the study. HI and YaO participated in the analytical plan, the interpretation of the results, and assisted in drafting the manuscript.

MaK participated in the design of the study. HK assisted with the interpretation of the results and helped draft the manuscript. All authors read and approved the final manuscript.

## Pre-publication history

The pre-publication history for this paper can be accessed here:

http://www.biomedcentral.com/1471-244X/11/118/prepub
